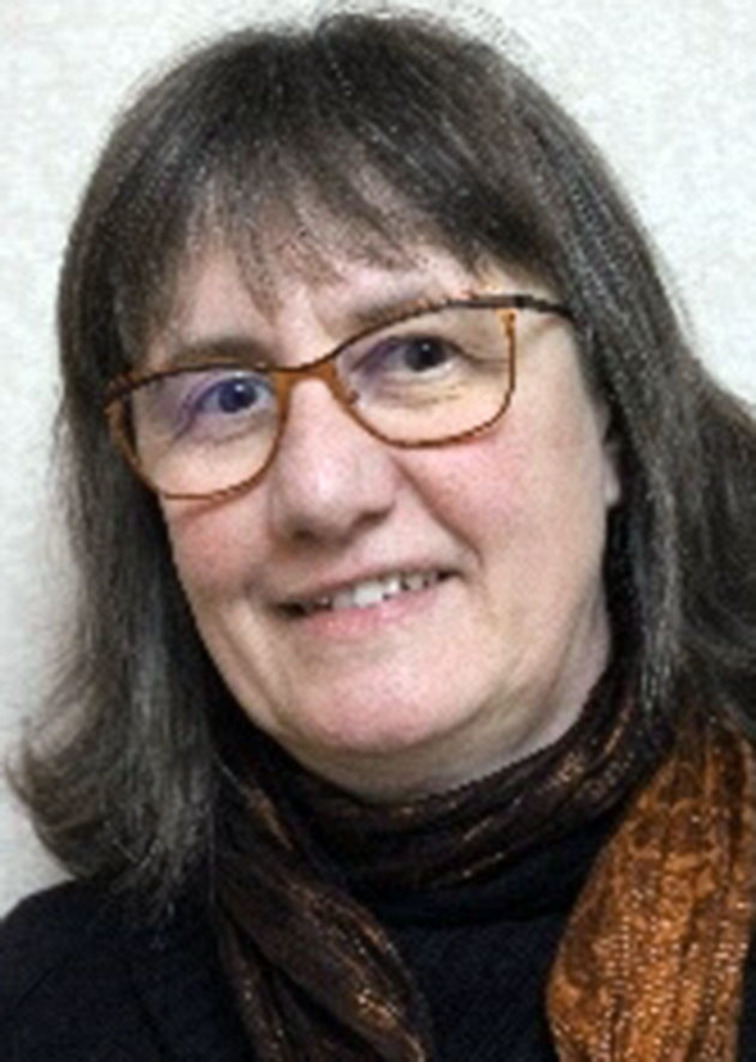# B4GALNT2 Controls Sd^a^ and SLe^x^ Antigen Biosynthesis in Healthy and Cancer Human Colon

**DOI:** 10.1002/cbic.202100363

**Published:** 2021-09-14

**Authors:** Sophie Groux‐Degroote, Dorothée Vicogne, Virginie Cogez, Céline Schulz, Anne Harduin‐Lepers

**Affiliations:** ^1^ Univ. Lille CNRS UMR 8576 UGSF Unité de Glycobiologie Structurale et Fonctionnelle 59000 Lille France

**Keywords:** colorectal cancer, glycoconjugates, human B4GALNT2, regulation, terminal glycosylation

## Abstract

The Sd^a^ carbohydrate antigen and the corresponding biosynthetic enzyme B4GALNT2 are primarily expressed in human normal colonic mucosa and are down‐regulated to variable degrees in colon cancer. On the other hand, the tumor associated antigen SLe^x^ is not detected in the healthy colon and is upregulated in colon cancer. High level of *B4GALNT2* gene expression appears to be a good marker of prognosis in colon cancer; however, the molecular mechanisms regulating these carbohydrate antigens’ expression are still poorly understood. We review here the most recent progress made towards understanding this balanced expression of blood group carbohydrate epitopes Sd^a^ and SLe^x^. In particular in recent years, we have attained a better understanding of genetic and epigenetic regulation of the *B4GALNT2* gene and of the subcellular fate of B4GALNT2 isoforms.

## Graphical abstract

**This review** summarizes the progress made in understanding the various regulatory mechanisms underpinning Sd^a^ and SLe^x^ expression in the human gastrointestinal tract.

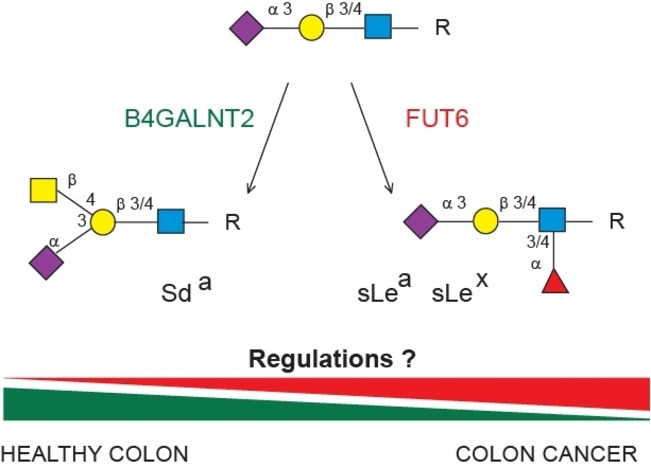

## 1 Introduction

**Scheme 1:**
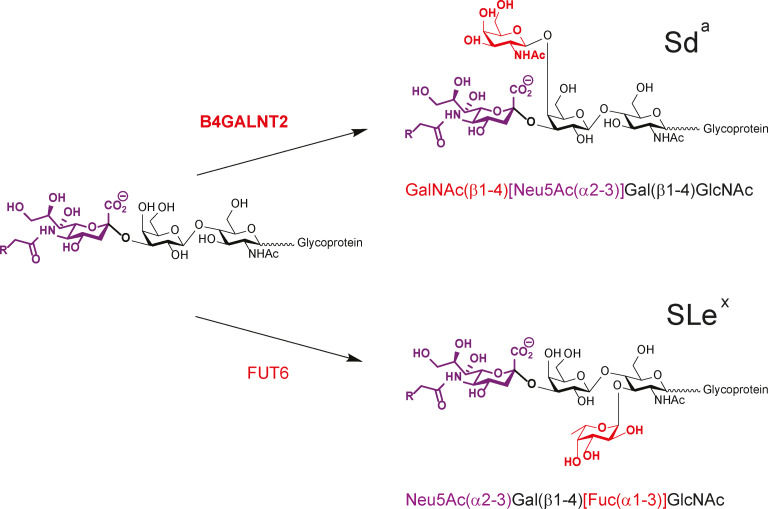
Last step of the biosynthetic pathways of Sd^a^ and SLe^x^ epitopes. On the left, the common sialylated saccharide which is used either by the B4GALNT2 or by the FUT6 enzyme for the synthesis of Sd^a^ or SLe^x^ epitopes.

Glycosylation is the most frequent and complex modification of lipids and proteins. It is relevant to every living cell, whose surface is covered by a carbohydrate layer known as glycocalyx. Glycosylation is variable according to organisms, cell types and physiological status of the cells and patterns are altered in multiple human diseases including congenital disorders of glycosylation, immune and inflammatory diseases, and cancer. Aberrant glycosylation is one of the most well‐known cancerous phenotypes in tumor cells and is considered as a hallmark of malignant transformation.^[1]^ These glycosylation changes of proteins include abnormal modifications of N‐glycans like core α1,6‐fucosylation, increased branching and α2,6‐sialylation, and modifications of O‐glycans like exposure of Tn antigen on mucin‐type O‐glycans.^[2]^ They affect the periphery of glycans (e. g. terminal glycosylation) and particularly sialylation thus having an impact in cell behavior, immune recognition, cell growth and cell interactions. Therefore, sialylated tumor‐associated carbohydrate antigens (TACA) like the sialyl Lewis x (SLe^x^) and Sd^a^ blood group determinants have attracted much attention these past twenty years. Intriguingly, the Sd^a^ epitope is primarily expressed in healthy colon, but not the SLe^x^ epitope, whereas Sd^a^ is dramatically downregulated in cancerous colon, whereas SLe^x^ is upregulated. The mechanisms that modulate the balanced expression of these sialylated TACA are not well understood.^[3]^ Of particular interest is the fact that Sd^a^ and SLe^x^ share a common biosynthetic pathway involving several α1,3‐fucosyltransferases (FUT3, FUT4, FUT5, FUT6, FUT7) and a unique β1,4‐*N*‐acetylgalactosaminyltransferase (B4GALNT2) (Scheme 1). FUT6 is the major α1,3‐fucosyltransferase involved in SLe^x^ biosynthesis in colon,^[4]^ however no upregulation of FUT6 or any other fucosyltransferases could be detected in colon cancer.[^3c^, ^5^] Therefore, the decrease in B4GALNT2 expression is responsible of the SLe^x^ expression in colon.^[3c]^ This review focusses on the human B4GALNT2 and comprehensively summarizes the main recent findings on the roles of this gene in the control of Sd^a^ and SLe^x^ antigens biosynthesis in healthy and cancer human colon.

## 2 The Sd^a^ Determinant

### 2.1 The Sd^a^ structures

**Figure 1:**
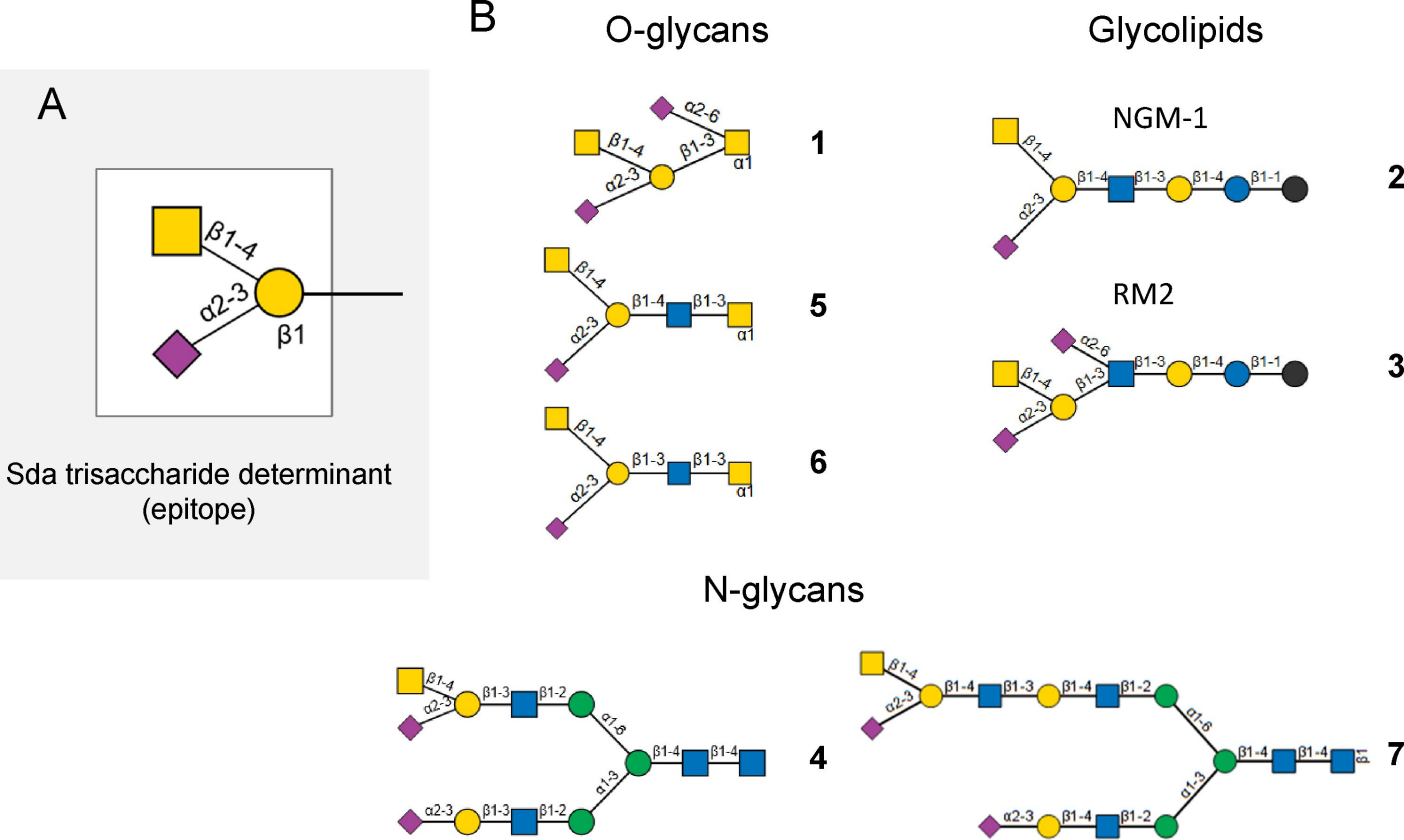
Structures bearing the Sd^a^ determinant. A) Sd^a^ trisaccharide epitope is composed of GalNAcβ1‐4[Neu5Acα2‐3]Galβ1‐. B) Structures bearing the Cad or Sd^a^ epitopes. Structure **(1)** is the Cad structure described on a core 1 *O*‐linked pentasaccharide of glycophorin A; structure **(2)** is the glycolipid sialylparagloboside with the epitope recognized by the anti‐NMG‐1 antibody; structure **(3)** is the disialylated glycolipid with the epitope RM2; structure **(4)** is the Sd^a^ bearing type 1 LacNAc **N**‐glycan identified in the serum of patients with gastric cancer; structures **(5)** and **(6)** are core 3 *O*‐glycans found on mucins of the healthy digestive tract; structure **(7)** is the Sd^a^ bearing type 2 LacNAc *N*‐glycan identified on the THGP.

The carbohydrate Sd^a^ antigen was described half a century ago^[6]^ on red blood cells (RBC) as the unique antigen of the blood group Sid. This is a high frequency epitope found on the erythrocytes of 90 % of Caucasian individuals. The Sd^a^ antigen is also detected in human adult tissues, mostly in the digestive tract (colon, kidney, stomach) and it is associated to soluble molecules in body fluids (urine, milk, meconium, and saliva) of 96 % of Caucasian individuals. Therefore, only 4 % are true Sd^a−^ individuals potentially producing anti‐Sd^a^ antibodies.[^3a^, ^7^] Intriguingly, in addition to these regular Sd^a+^ and Sd^a−^ phenotypes, a Sd^a++^ (or Cad or super‐Sid) phenotype was described as a very reactive antigen that reacted strongly with anti‐ Sd^a^ antibodies.^[8]^ The chemical structure of the Sd^a^ determinant was found to be the sialylated trisaccharide GalNAcβ1‐4[Neu5Acα2‐3]Galβ1‐ illustrated in Figure 1A. It was elucidated in RBC of Cad individuals, where the major Cad structure was found on an O‐linked pentasaccharide of glycophorin A (structure **(1)**, Figure 1B), which is not found in Sd^a+^ and Sd^a−^ individuals.^[9]^ Furthermore, in Cad and Sd^a+^ individuals RBC, the glycolipid sialylparagloboside could be also modified with a β1,4‐linked GalNAc residue, forming the GalNAcβ1‐4[Neu5Acα2‐3]Galβ1‐4GlcNAcβ1‐3Galβ1‐4Glc‐ceramide (structure **(2)**, Figure 1B).^[10]^ In the human fetus, very low levels of Sd^a^ antigen are detected in RBC and colon. However, it has been detected in the urine and at high levels in the saliva of the newborn infant. The Sd^a^ antigen appears on the RBCs later on, between 10 weeks to 7 months.[^6^, ^7^, ^11^] Although the immunodominant monosaccharide was found to be the GalNAc residue β1,4‐linked to a galactose (Gal) residue,^[12]^ there is still a lack of information on the underlying carbohydrate structures and Sd^a^/Cad carriers that could explain these various Sd^a^/Cad activities reported in serum.

In human adult tissues, the Sd^a^ determinant was identified as part of the glycolipid GalNAc disialosyl Lc4Cer (IV4GalNAcIV3NeuAcIII6NeuAcLc4, GalNAcDSLc4, GalNAcβ1‐4[Neu5Acα2‐3]Galβ1‐4[Neu5Acα2‐6]GlcNAcβ1‐3Galβ1‐4Glc‐ceramide, structure **(3)**, Figure 1B) in the renal carcinoma TOS‐1 and prostate cancer cell lines LNCaP and PC‐3.^[13]^ The disialylated hexasaccharide epitope (GalNAcβ1‐4[Neu5Acα2‐3]Galβ1‐3[Neu5Acα2,6]GlcNAcβ1‐3Galβ1‐), also known as RM2‐epitope because it is highly reactive to the RM2 antibody, correlated with high metastatic potential of RCC^[14]^ and was synthetized for further vaccine development.^[15]^ Recent glycomics studies have led to the identification of N‐glycans carrying type 1 LacNAc structures (Galβ1‐3GlcNAc) with Sd^a^ determinant in the serum of patients with gastric cancer (structure **(4)**, Figure 1B).^[16]^ Since no upregulation of Sd^a^ synthesis could be detected in gastric cancer patient, an accelerated release of these Sd^a^ bearing structures in the blood stream was suggested. The Sd^a^ determinant is also a major structural feature encountered in the mucus layer of the human descending colon. It has been described on the borders of epithelial cells and in goblet cells. The Sd^a^ carriers are the highly glycosylated mucins (e. g. MUC2), on which the Sd^a^ epitope is mostly found on core 1 and core 3 O‐glycans (structures **1**, **5** and **6** in Figure 1B).^[17]^ In the urine of Sd^a+^ individuals, the abundant uromodulin, also known as Tamm‐Horsfall glycoprotein (THGP),^[18]^ carries the antigen on N‐glycans with two LacNAc repeats (Galβ1‐4GlcNAc) as illustrated in Figure 1B (structure (**7**)).[^12^, ^19^]

### 2.2 Molecular tools to detect Sd^a^ antigen

Several plant lectins with GalNAc‐binding specificity like the *Dolichos biflorus* (DBA) and *Helix pomatia* (HPA) agglutinins and the B4 lectin from *Vicia villosa* (VVA) seeds were used for the agglutination of Cad erythrocytes[^8a^, ^20^] and the detection of the Sd^a^ and Cad antigens. However, these lectins are not specific for the GalNAc residue found in the Sd^a^ determinant since they also detect the GalNAc residue in the Forssman disaccharide (GalNAcα1‐3GalNAc), the blood group A determinant (GalNAcα1‐3[Fucα1‐2]Galβ1‐) and LacdiNAc (GalNAcβ1‐4GlcNAc) epitope.^[21]^

Several monoclonal antibodies (mAbs) have been developed that recognize the Sd^a^ determinant. The mouse CT1 and CT2 mAbs recognized the Sd^a^ antigen on mouse cytotoxic T lymphocytes.^[22]^ Interestingly, CT1 recognizes a Neu5Ac‐containing Sd^a^ epitope, whereas CT2 recognizes a Neu5Gc‐containing Sd^a^ epitope.^[23]^ The human KM531^[24]^ and KM694^[25]^ mAbs were raised against the G_M2_ ganglioside (GalNAcβ1‐4[Neu5Acα2‐3]Galβ1‐4Glc‐Cer), which also shows the Sd^a^ determinant. Therefore, similarly to the Sd^a−^ or Cad‐specific lectins, these four mAbs bind to GalNAc present in other potential antigens, as for G_M2_. Of particular interest, these mAbs have a higher affinity for the Sd^a^ determinant found on either glycoproteins or glycolipids like the NGM‐1 epitope (sialylparagloboside, structure **(2)**, Figure 1B) as compared to GM2. However, the HCM31 (human colorectal mucin 31) mAb recognized the Sd^a^ antigen in goblet cells mucins, but did not bind G_M2_.^[26]^

## 3 The Human *B4GALNT2* Gene

### 3.1 The human *B4GALNT2* gene organization

The biosynthesis of Sd^a^ and Cad antigens is driven by a unique gene, the human *B4GALNT2* gene (HGNC:24136); this is the official human gene symbol according to the human gene nomenclature committee HUGO (http://www.genenames.org) of this gene previously known as *GALGT2*. The human gene cloned from colonic cancer Caco‐2 cells includes 11 coding exons and was mapped onto chromosome 17q21.33.^[27]^ Until recently, the genetic basis of Sd^a^ was not solved and therefore was classified in the 901 series of high incidence antigens. In 2019, the sequencing of the *B4GALNT2* gene in nine Sd^a−^ individuals with anti‐ Sd^a^ antibodies in their serum identified the single nucleotide polymorphism (SNP) rs7224888:T>C in exon 10 of 6 individuals as highly associated with the phenotype.^[28]^ This SNP leads to a missense mutation, which likely disrupts the folding of the enzyme and abolishes its activity. Therefore, the International Society of Blood Transfusion (ISBT) Working Party for Red Cell Immunogenetics and Blood Group Terminology moved the Sd^a^ antigen from the series of high‐frequency antigens to its own blood group system SID, number 038.^[28]^ However, the genetic backgrounds of a minority of Sd^a−^ and Cad phenotype still remain unknown.

**Figure 2:**
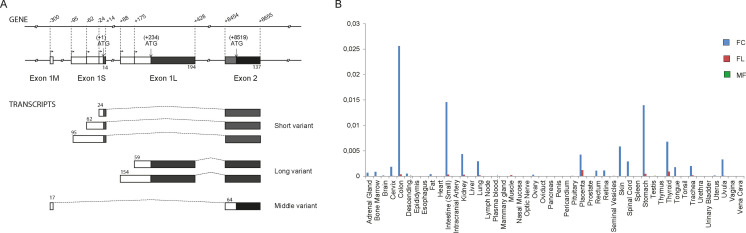
The human *B4GALNT2* gene 5’ end organization and expression. A) *B4GALNT2* 5’‐Untranslated region (UTR) were detected using 5’ end amplification of cDNA ends (5’RACE) approach. The three types of *B4GALNT2* transcripts are generated through the use of three distinct alternative first exons (1M, 1S and 1L). The three different NH2 termini are indicated by black or grey boxes and the untranslated region by white boxes. B) The expression of the three types of transcript of the human *B4GALNT2* gene was examined by Q‐PCR in various human tissues using primer pairs sitting in the three distinct exons 1M, 1S and 1L, respectively. The expression of each transcript was normalized to that of *HPRT*.

Northern blot analysis indicated the existence of at least five *B4GALNT2* transcripts mainly expressed in colon and also to much lower levels in ileum, stomach and kidney.^[27b]^ Further PCR analyses suggested divergences in the 5’‐ and 3’‐untranslated region (UTR) of the human *B4GALNT2* transcripts, that sometimes presented very long 3’‐ends.^[27]^ Interestingly, *B4GALNT2* possesses several alternative first exons (AFE), a feature that is unique to the human *B4GALNT2* gene and suggests complex transcriptional regulation in human tissues (Figure 2A). More recently, 5’‐RACE analyses in human colon have shown that the *B4GALNT2* gene contains three AFE and three sets of transcriptional start sites (TSS): the long exon 1 (exon 1L) is 253 to 348 nucleotides (nt) long, while the short exon 1 (exon 1S) is 38 to 109 nt long and the middle exon 1 (exon 1M) is 51 nt long[^3c^, ^27b^] (Wavelet et al. 2021, accepted in BBGRM). These three types of *B4GALNT2* transcripts are differentially expressed in human tissues (Figure 2B). However, the SF‐transcript variant is the one that is primarily expressed in the digestive tract (colon, small intestine, stomach, and kidney) and to a lesser extent in placenta, skin, spinal cord, thyroid and trachea. The FL‐transcript variant is detected in placenta, thyroid and at low levels in the digestive tract, whereas the MF‐transcript variant is almost not detected.

### 3.2 The human *B4GALNT2* gene expression profile in digestive tract

Seminal studies established that the Sd^a^ determinant is found on the glycosphingolipid sialylparagloboside in the healthy stomach and is associated to mucin‐type *O*‐glycosylproteins in the healthy colon.[^24^, ^29^] Interestingly, the Sd^a^ carbohydrate structure is strongly decreased in cancer colon tissues[^3c^, ^30^] and completely lost in gastrointestinal cancer cell lines such as Kato III, HCT‐116 or LS174T cells.[^3c^, ^31^] The absence of Sd^a^ determinant in gastric and colon cancer was due to decreased expression of the *B4GALNT2* gene.^[32]^ Interestingly, the forced expression of *B4GALNT2* in various gastric (Kato III) and colorectal (HT29, LS174T) cancer cells resulted in the replacement of SLe^x^ by Sd^a^ determinants suggesting that the two epitopes could be carried on the same molecules and also reduced the metastatic potential of cancer cells.[^25^, ^33^] High SLe^x^ expression, ligand for E‐selectin, correlates with advanced stages and metastasis in colorectal cancer (CRC) and gastric cancer, and reduced SLe^x^ ‐selectin interactions could explain the reduced metastatic properties of *B4GALNT2*‐transfected LS174T and Kato III cells. However, in colon samples, the Sd^a^ epitope is found on very high molecular weight proteins, whereas SLe^x^ is found on both mucin‐type and lower molecular weight glycoproteins, an observation that does not support full competition between the two antigens for the same site.^[3c]^ Recently, the SF‐transcript variant was described to be dramatically decreased in CRC, whereas the LF‐ and MF‐variants were constantly expressed at very low levels, whatever the pathological state, further suggesting complex transcriptional regulatory mechanisms modulating the SF‐transcript variant expression.^[3c]^

**Figure 3:**
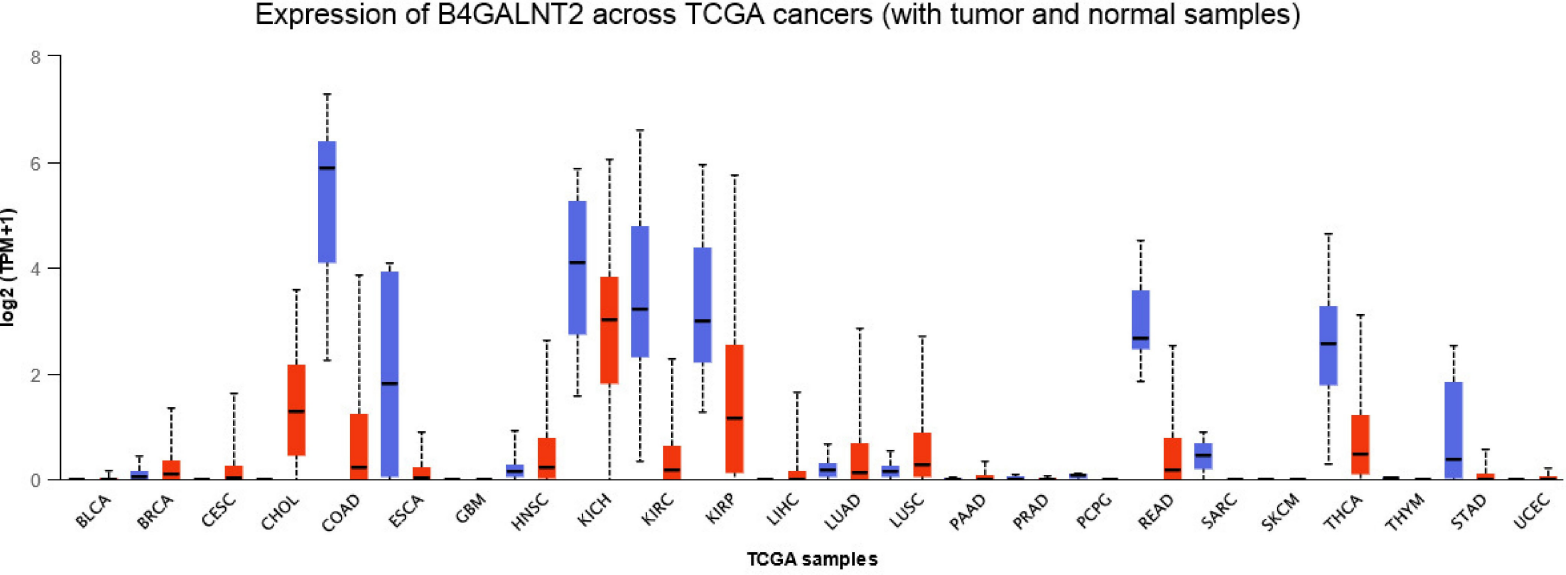
Expression of *B4GALNT2* gene across TCGA cancers (with tumor (red) and normal (blue) samples). The UALCAN platform analysis (http://ualcan.path.uab.edu) was used to establish the pan cancer profile of expression of the *B4GALNT2* gene in various human tumors.^[35]^ The abbreviation of Colon Adenocarcinoma is COAD; the other TCGA abbreviations are given at the web site https://gdc.cancer.gov/resources‐tcga‐users/tcga‐code‐tables/tcga‐study‐abbreviations.

These past years, high‐throughput technologies generated a huge amount of human gene expression data gathered in four major data resources: The Cancer Genome Atlas (TCGA), Genotype‐Tissue Expression (GTEx), Cancer Cell Line Encyclopedia (CCLE) and MD Anderson Cell Lines Project (MCLP). Web‐based tools were developed like Gene Expression Profiling Interactive analysis (GEPIA)^[34]^ or UALCAN^[35]^ for effective cancer data online analyses. In this study, UALCAN was used to visualize the expression profile of *B4GALNT2* across various TCGA cancers and paired‐normal samples (Figure 3). *B4GALNT2* is highly expressed in the digestive tract and thyroid tissues and it appears to be significantly downregulated in COlon ADenocarcinoma (COAD), in KIdney CHromophobe, KIdney Renal clear Cell Carcinoma, KIdney Renal Papillary Cell Carcinoma (KICH, KIRC and KIRP), STomach ADenocarcinoma (STAD) and REctal ADenocarcinoma (READ), and ESophageal CArcinoma (ESCA) and THyroid CArcinoma (THCA). Recent TCGA analyses made by Pucci and collaborators indicated that CRC patients with higher expression levels of *B4GALNT2* had longer survival than those who did not express *B4GALNT2* further suggesting that *B4GALNT2* could be a marker of good prognosis for CRC patients.^[36]^ However, the benefits of *B4GALNT2* expression in colonic tissues cannot be reduced to its impact on SLe^x^ expression: stable transfection of SLe^x^ negative colon cancer cell lines SW480 and SW620 by *B4GALNT2* cDNA resulted in the down‐regulation of cell malignant properties, with decreased growth in soft agar and spheroid formation, and also in profound modifications of the transcriptome. Some genes were impacted in a cell type specific manner, and there were also genes whose expression was modified in both cell lines.^[36a]^ Genes whose expression was either down‐ or up‐regulated by *B4GALNT2* were related to apoptosis, cell adhesion, cell cycle, cytoskeleton and cytokinesis, with a marked decreased expression of the stemness marker CD44 and a down‐regulated ALdehyde DeHydrogenase (ALDH) expression in both SW480 and SW620 cells. Consequently, B4GALNT2 seems to inhibit both malignant properties and stemness of colon cancer cells, independently of SLe^x^ expression.

### 3.3 Epigenetic and genetic regulation of the human *B4GALNT2* gene

The upstream genomic regulatory regions surrounding the three AFE 1M, 1L and 1S of the *B4GALNT2* gene are rich in CpG islands indicating that DNA methylation could contribute to the regulation of *B4GALNT2* expression in CRC. Indeed, *B4GALNT2* promoter methylation was observed in gastric cancer cases and in the majority of gastric and CRC cell lines analyzed in the Kawamura and collaborators studies.^[37]^ However, anti‐DNA methylation treatment in cell lines with the methylation inhibitor 5‐aza‐2’‐deoxycytidine could induce only a weak expression of the *B4GALNT2* gene and Sd^a^ antigen in these cells^[37a]^ indicating that other regulatory mechanisms are likely involved. In a recent study, the CRC genomic and epigenetic data from the TCGA database were integrated to characterize the DNA methylation landscape of the *B4GANT2* gene.^[36b]^ These analyses showed no major differences of DNA‐methylation pattern between the upstream and downstream genomic regions of the *B4GALNT2* locus in normal and tumor tissues suggesting that these changes would not be responsible for the downregulated expression of *B4GALNT2* in CRC. Of particular interest, this study also pointed to the correlation between high *B4GALNT2* expression and high DNA methylation of an intronic site (cg043380107) located between exon 6 and exon 7, and reduced *B4GALNT2* expression for low methylation at this site.^[36b]^ Other epigenetic modifications including histone modifications like acetylation and methylation were also investigated. Kawamura and collaborators obtained evidence using butyrate, a histone deacetylase inhibitor, that these histone modifications are not involved in the human *B4GALNT2* gene regulation.^[37a]^

**Figure 4:**
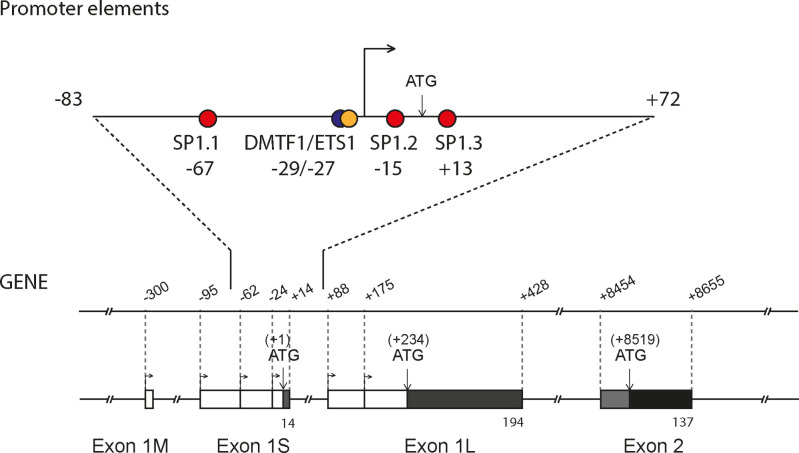
Promoter analysis of the human *B4GALNT2* gene. Delineation studies indicated that the minimal core promoter of human *B4GALNT2* is restricted to a −72/+83 region upstream the SF‐translational start site (+1/ATG). Different approaches (bioinformatic analyses, site‐directed mutagenesis and luciferase assays, siRNA/overexpression strategies of TF, electrophoretic mobility shift assays (EMSA) and ChIP experiments suggested that transcription factors SP1, ETS1 and DMTF1 were implicated in the promoter regulation of the *B4GALNT2* gene (Wavelet et al. 2021, accepted in BBGRM).

As a first step to address the complex issues of control mechanisms underpinning Sd^a^ expression in the human gastrointestinal tract, we started to dissect the molecular genetic mechanisms of regulation of *B4GALNT2* (Wavelet et al. 2021, accepted in BBGRM). Various constructions were made with the Luciferase reporter gene including potential genomic regulatory sequences. These constructs were transiently transfected in gastric and colonic cells as well as non‐colonic cells. Luciferase activities assays enabled us to propose the existence of two promoter regions upstream each first exon 1S and 1L. However, none of these genomic regions could explain the colon specific expression of *B4GALNT2*. A minimal basal promoter was delineated in the −83/+72 nucleotides (nt) region relative to the translational start site present in the exon 1S of *B4GALNT2* (Figure 4). In this region, several Cis‐acting elements were identified including ETS1, DMTF1 and SP1 binding sites that were functionally characterized using site directed mutagenesis, Luciferase assays, Chromatin Immuno‐Precipitation (ChIP) assays and Electrophoretic Mobility Shift Assay (EMSA) experiments. It was shown that *B4GALNT2* transcriptional activation mostly required ETS1 combined to DMTF1 and to a lesser extent SP1, transcription factors that also play a role in cell invasion and carcinogenesis (Wavelet et al. 2021, accepted in BBGRM). A gene regulatory network analysis showed that several glyco‐genes belonging to the last steps of *O*‐glycosylation pathway including sialyltransferases and mucin genes are likely regulated by the same transcription factors (Wavelet *et al*., submitted data).

**Figure 5:**
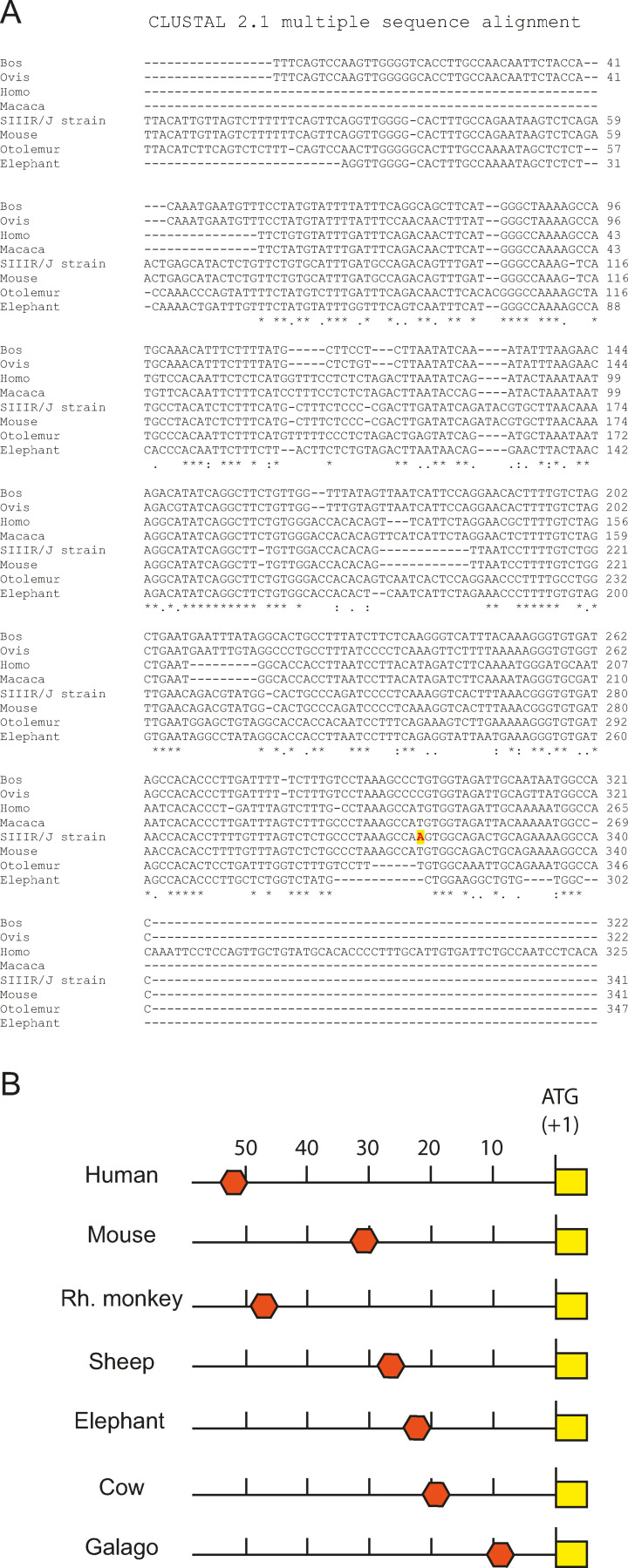
Potential conserved enhancer region in the mammalian *b4galnt2* gene. A) Blast analysis at NCBI and multiple sequence analysis of the 400 bp conserved genomic region. B) Schematic representing the various localization of the 400 bp region found upstream the *b4galnt2* locus at 9,623 bp in the small eared galago (*Otolemur garnettii*), 19,046 bp in the cattle (*Bos taurus*), 22,671 bp in the elephant (*Loxodonta africana*), 27,654 bp in the sheep (*Ovis aries*), 47,610 bp in the Rhesus monkey (*Macaca mulatta*), 50,276 bp in the human (*Homo sapiens*) and at 30,571 bp in the mouse (*Mus musculus*).

This first piece of work cannot fully explain the molecular mechanisms underpinning Sd^a^ expression in the healthy colon and its disappearance in the cancerous colon to the benefit of SLe^x^. Beyond promoter region and transcription factors, other regulatory mechanisms are likely involved like long‐range regulatory elements with enhancer motifs that could explain the tissue‐specific expression of *B4GALNT2* gene. Interestingly, the mouse strain RIIIS/J shows a conserved ∼400 base pair (bp) region located about 30 kb upstream the *b4galnt2* gene containing a mutation responsible for low plasma levels of the von Willebrand Factor (vWF). It was shown that this mutation led to the expression of *b4galnt2* in the vascular endothelium and its disappearance in colon.^[38]^ A BLAST analysis of this 400 bp region in various mammalian genomes led to its identification at various locations upstream *b4galnt2*. It is found at 9,623 bp upstream the b4galnt2 locus in the small eared galago (*Otolemur garnettii*), 19,046 bp in the cattle (*Bos taurus*), 22,671 bp in the elephant (*Loxodonta africana*), 27,654 bp in the sheep (*Ovis aries*), 47,610 bp in the Rhesus monkey (*Macaca mulatta*), 50,276 bp in the human (Homo sapiens) and at 30,571 bp in the mouse (*Mus musculus*) (Figure 5). Other transcriptional regulatory mechanisms of *B4GALNT2* could involve downstream genomic regions and non‐coding RNA (ncRNA). The 1,352 bp sense intronic ncRNA gene Lnc RP11‐708H21.4 also called Lnc‐ABI3‐6 : 1 gene (ID: NONHASAT054511.2 in the NONCODE database; gene ID: 192343 in NCBI gene division), located in the 17q21 corresponds to a sequence in the 3‐untranslated region of *B4GALNT2*. Interestingly, it is downregulated in CRC and its upregulation inhibits migration and invasion and induces 5‐FU sensitivity in CRC.^[39]^

## 4 B4GALNT2 Proteins

### 4.1 The three B4GALNT2 protein isoforms

The first evidence of the existence of an β1,4‐*N*‐acetylgalactosaminyltransferase catalyzing the transfer of GalNAc from uridine diphosphate (UDP)‐GalNAc to the sialylated *N*‐glycans of the major urinary Tamm‐Horsfall glycoprotein was obtained in the eighties by the group of F. Serafini‐Cessi in guinea pig kidney^[40]^ and by Piller and collaborators in human kidney.^[41]^ The human enzyme also showed activity towards elongated glycosphingolipids like sialylparagloboside, but had no activity towards ganglioside G_M3_.

**Figure 6:**
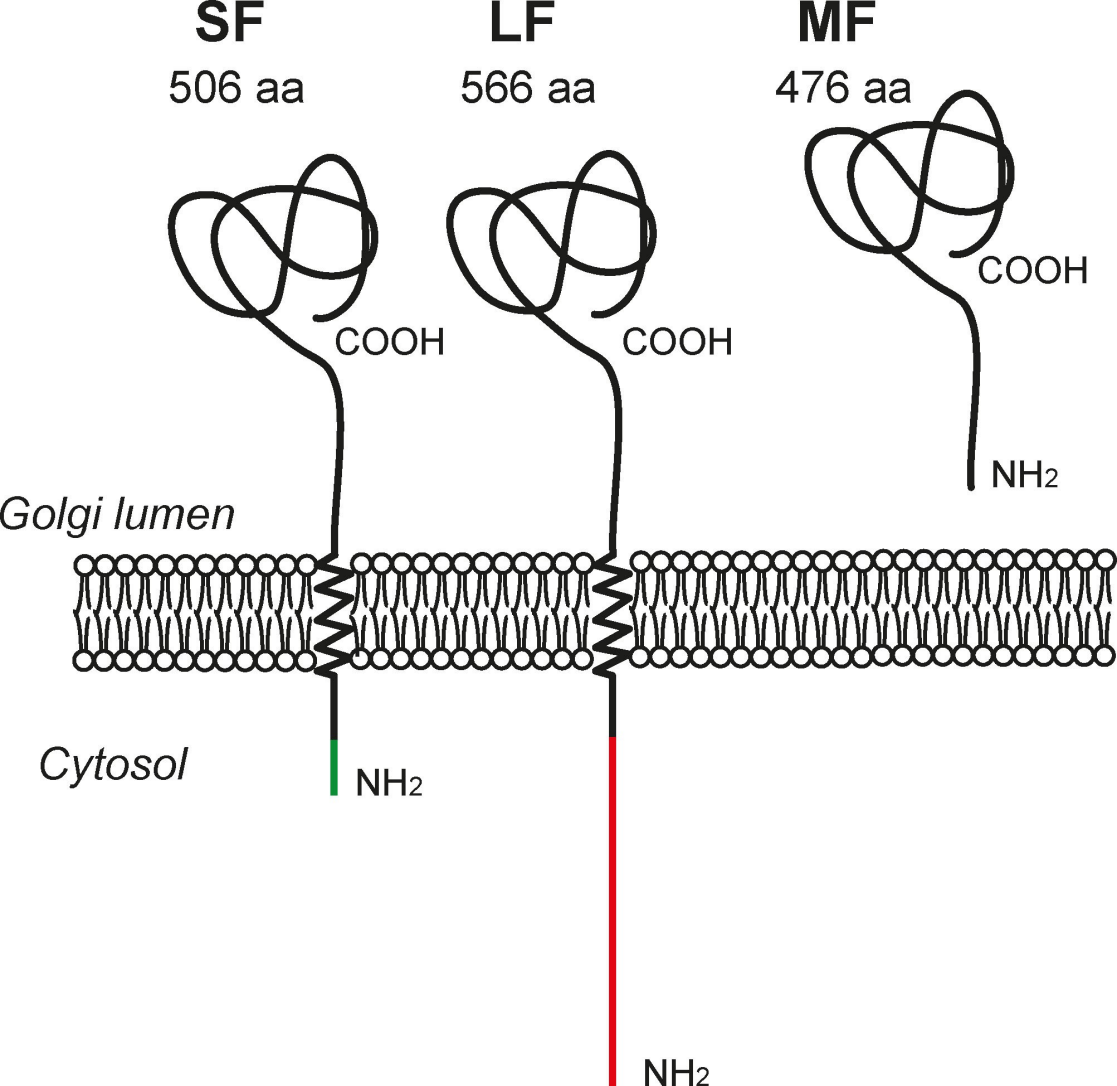
The various B4GALNT2 protein isoforms translated and detected in the human healthy colon. From left to right, the short protein isoform (SF‐isoform), the long protein isoform (LF‐isoform) showing an unusual extended cytoplasmic tail and the soluble middle protein isoform (MF‐isoform) are represented in the Golgi apparatus of cells.

Interestingly, since both first exons 1L and 1S contain a translational start site, the human *B4GALNT2* gene gives rise to at least two different transmembrane protein isoforms with different amino‐terminal regions: a 566 amino‐acid (aa) long isoform (LF) with a very long cytoplasmic tail and a 506 aa short isoform (SF) with a short cytoplasmic tail (Figure 6). The third transcript variant including exon 1M is predicted to generate a soluble form (MF) of the enzyme since exon 1M lacks a translational start codon. Two in frame ATG are located in exon 2 and would give rise to a 476 or 480 aa isoform with no transmembrane region since these start codons are located at the end of the predicted transmembrane domain (Figure 6). Therefore, the three transcript variants encode three B4GALNT2 protein isoforms, a short, long and middle forms (SF, LF and MF, respectively) sharing the same catalytic and stem domain in the Golgi lumen. Accordingly, expression of the three protein isoforms could be detected to variable levels in human tissues samples by Western Blotting using a specific mAb directed towards the common stem region of the three B4GALNT2 isoforms. The short B4GALNT2 isoform is the one that is predominantly expressed in the healthy colon tissue and normal colonic cell line CcD 841 CoN and which is down‐regulated in colon cancer and cancer cell lines HCT‐116 and LS174T.^[3c]^

### 4.2 Differential subcellular localization of the B4GALNT2 isoforms

**Figure 7:**
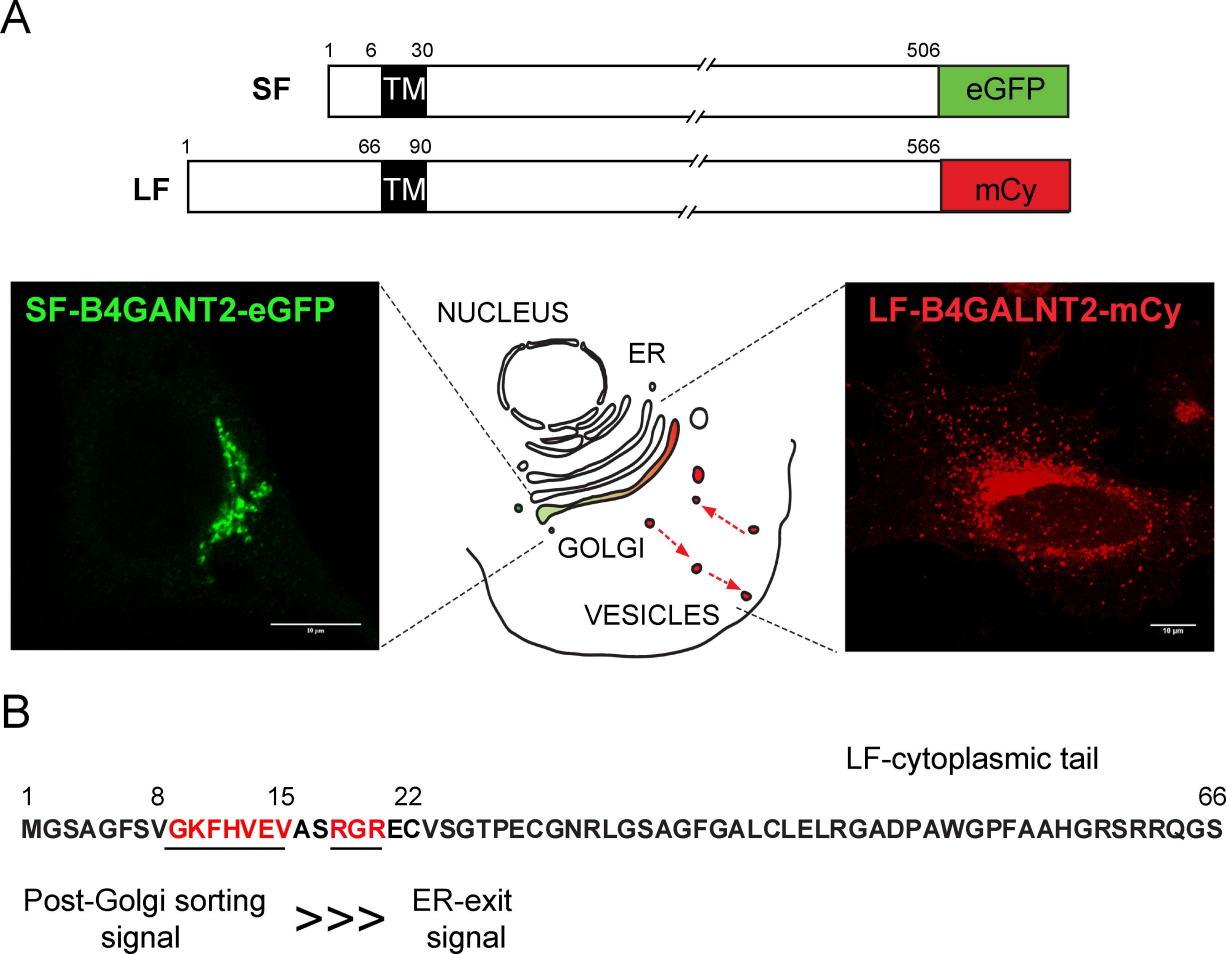
Immunofluorescence studies using confocal microscopy. A) Schematic representation of the chimeric constructs used. The SF‐isoform tagged with eGFP (green) localizes in the trans‐Golgi of transfected HeLa cells and the LF‐isoform tagged with mCherry (red) localizes in the trans‐Golgi and in post‐Golgi vesicles. B) Two signals were detected in the cytoplasmic tail of the LF‐isoform: an ER‐exit signal RGR (aa 18–20) and a post‐Golgi sorting signal the heptapeptide GKFHVEV (aa 9–15). This latter signal is very strong and targets LF‐B4GALNT2 to vesicles even in the absence of the ER‐exit signal, which is symbolized here by ⋙.

To study their subcellular localization, the two main B4GALNT2 isoforms SF and LF were produced as recombinant chimeric fluorescent proteins in various human cultured cells. The SF was tagged with the green fluorescent protein (e‐GFP) whereas the LF was endowed with m‐cherry (mCy) (Figure 7A) and transiently transfected in HeLa cells. The chimeric proteins were observed using confocal microscopy and detected in the trans‐Golgi network as ascertained using markers of these secretory pathway compartments like TGN46 and TMEM165.^[42]^ Of particular interest, the LF isoform was also largely distributed in post‐Golgi vesicles, which is very unusual for a glycosyltransferase (Figure 7A). However, no overlap could be detected with the endosomal marker Rab11 A, the Early Endosomal Antigen‐1 marker (EEA1) or the Lysosome‐Associated Membrane Protein 2 marker (LAMP2) and the nature of LF‐B4GALNT2 containing vesicles remains to be determined.^[42]^

The molecular mechanism by which the LF‐isoform is targeted to these post‐Golgi vesicles was dissected. Several deletion constructs devoid of the first 2–22, 2–8, 9–15, 16–22, 23–43, 44–64 aa were made, tagged with mCherry and transiently transfected in HeLa cells. These experiments provided evidence that the first 2–22 region of the LF‐isoform cytoplasmic tail contains several strong and critical signals for the LF‐B4GALNT2 fate in the cell (Figure 7B). A dibasic signal RGR motif at positions 18–20 was essential for ER exit and moreover, a dominant and strong GKFHVEV signal at positions 9–15 was crucial for both ER exit and post‐Golgi subcellular localization.^[42]^

## 5 Conclusions

The recent studies conducted in the field started to shed light on the mechanisms by which tissue and stage‐specific expression of B4GALNT2 and Sd^a^ is achieved in the human digestive tract. Epigenetic mechanisms like DNA methylation and genetic mechanisms involving ETS1, SP1 and DMTF1 TF are likely involved although they cannot alone not account for the selective and the high level of Sd^a^ expression in the colon. Each B4GALNT2 protein isoform (SF and LF) is primarily found in the trans‐Golgi network of cells. In addition, the LF‐protein isoform actively localizes to post‐Golgi vesicles thanks to the presence of a strong aa signal present in its cytoplasmic tail. Such an unusual subcellular localization could confer novel functions to this LF‐isoform in specific organs/cell types or at specific developmental stages. Influence of additional post‐translational modifications of the B4GALNT2 isoforms like N‐glycosylation in the luminal domain of these enzymes and phosphorylation/O‐GlcNAcylation of the extended LF‐cytoplasmic tail is currently under investigation to better understand the multiple levels of Sd^a^ regulated expression in the digestive tract.

## Conflict of interest

The authors declare no conflict of interest.

## Biographical Information

*Dr. Sophie Groux‐Degroote obtained her PhD in Biochemistry and Molecular Biology in 1999 at the University of Lille (FRANCE). She has been Assistant Professor at the University of Lille since 2005. Her studies at the Structural and Functional Glycobiology Unit aim at deciphering the mechanisms by which terminal glycan structures are modified in pathologies like cancers and inflammatory diseases. She focusses on gangliosides as biomarkers and therapeutic targets in breast cancer*.

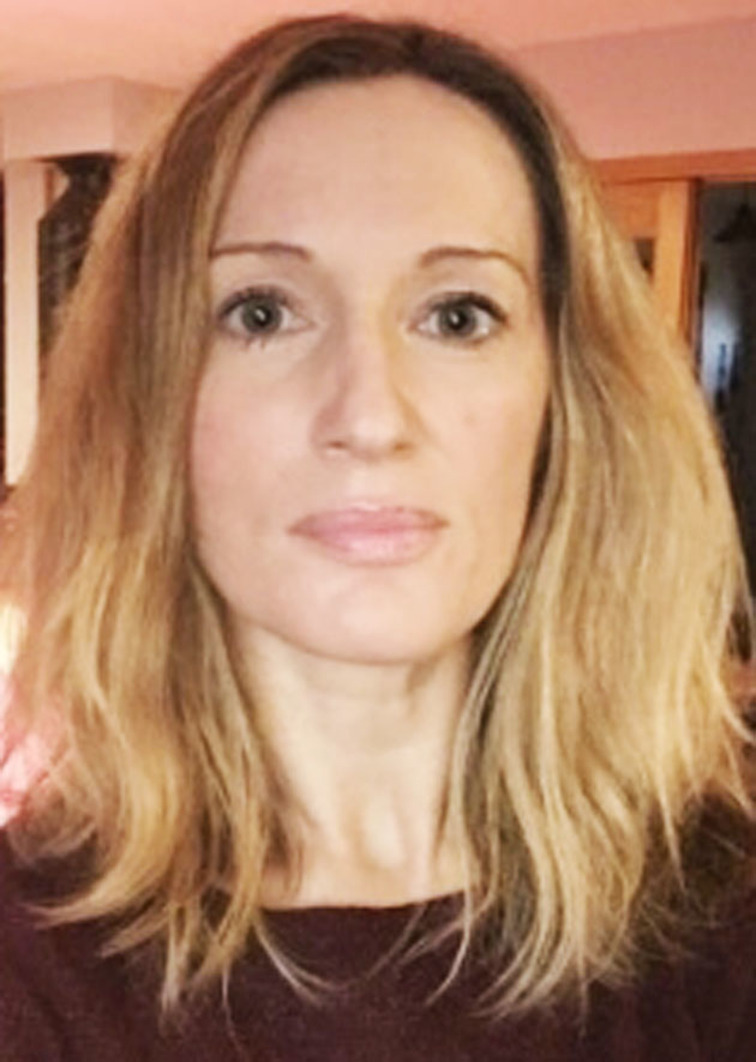

## Biographical Information

*Dorothée Vicogne obtained her Master's in Cellular and Molecular Biology at the University of Lille (FRANCE) in 2003. After four years at the University of Stony Brook (NY, USA) as Research Support Specialist, she became Engineer at the University of Lille in 2008. Her studies at the Structural and Functional Glycobiology Unit aim at deciphering the mechanisms by which terminal glycan structures are modified in pathologies like cancers. She focusses on sialylated carbohydrate antigen expression and the associated glycosyltransferases*.

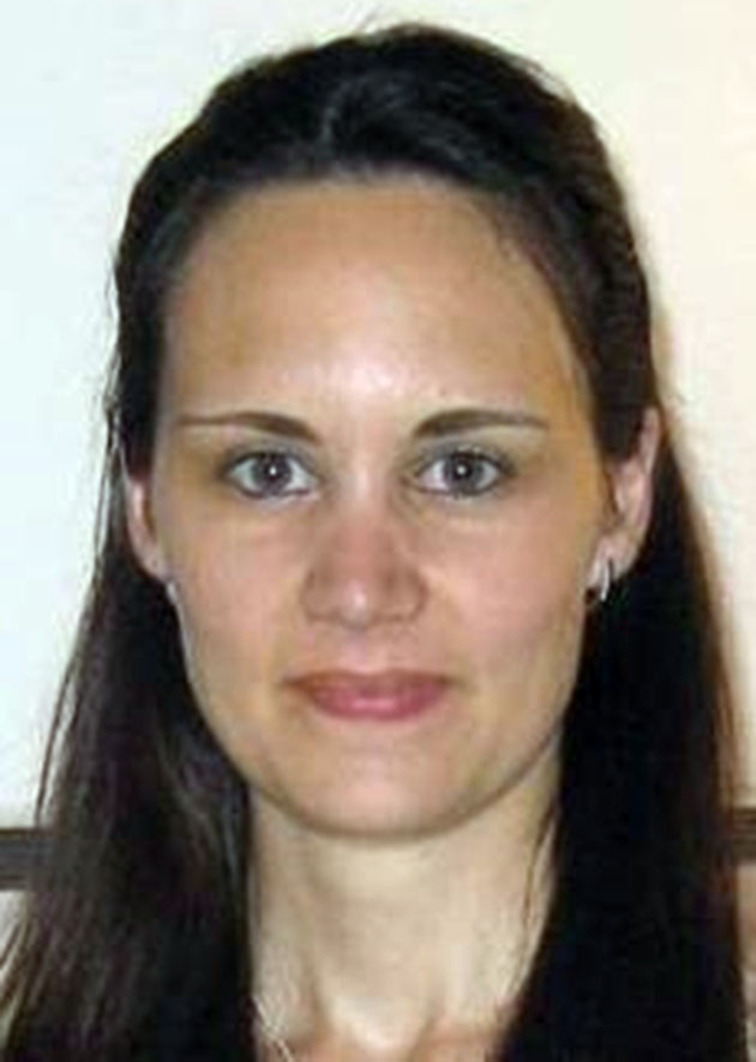

## Biographical Information

*Dr. Virginie Cogez obtained her PhD in Biology and Health Sciences in 2001 at the University of Lille (FRANCE). She has been Assistant Professor at the University of Lille since 2002. Her studies at the Structural and Functional Glycobiology Unit aim at deciphering the mechanisms by which terminal glycan structures are modified in pathologies like cancers. She focusses on structure/function relationships of glycosyltransferases*.

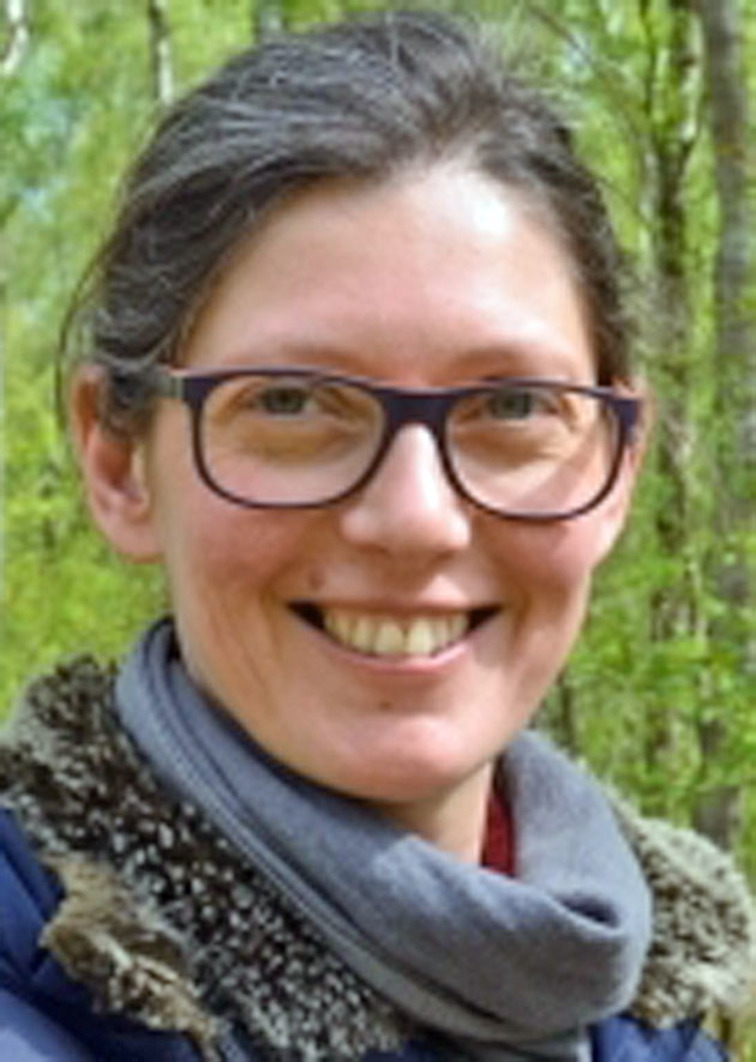

## Biographical Information

*Céline Schulz obtained her Master's in Cellular and Molecular Biology at the University of Lille (FRANCE) in 2011. She became an Engineer at CNRS in 2017. Her studies at the Structural and Functional Glycobiology Unit (University of Lille) aim at deciphering the mechanisms by which terminal glycan structures are modified in pathologies like cancers, congenital disorders of glycosylation and metabolic diseases*.

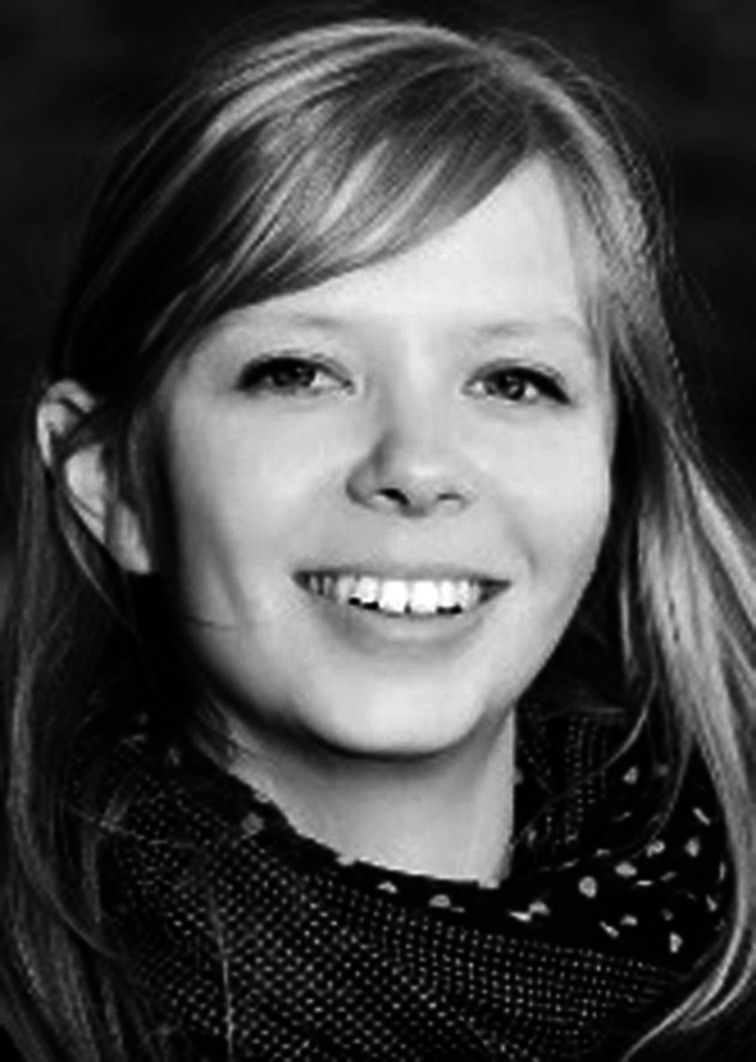

## Biographical Information

*Dr. Anne Harduin‐Lepers obtained her PhD in Life and Health Sciences in 1990 at the University of Lille (FRANCE). After a post‐doctoral stay at the Johns Hopkins Hospital of Baltimore (USA), she became a CNRS researcher in 1993, and since 2012 she has been a director of research. Her studies at the Structural and Functional Glycobiology Unit (University of Lille) aim at deciphering the mechanisms by which terminal glycan structures are synthetized and modified in pathologies like cancers or congenital disorders of glycosylation. Her interests focus on structure/function relationships of glycosyltransferases and their evolutionary relationships in Metazoa*.